# Supplementary material for: Comparative multiomics analysis of cell physiological state after culture in a basket bioreactor
Source: Sci Rep. 2022 Nov 23;12:20161. doi: 10.1038/s41598-022-24687-4 (PMC9686226; doi:10.1038/s41598-022-24687-4)

3666048.1:65014682:65014802:+@NW_023666048.1:65018846:65018885:+@NW_023666048.1:65019044:6501

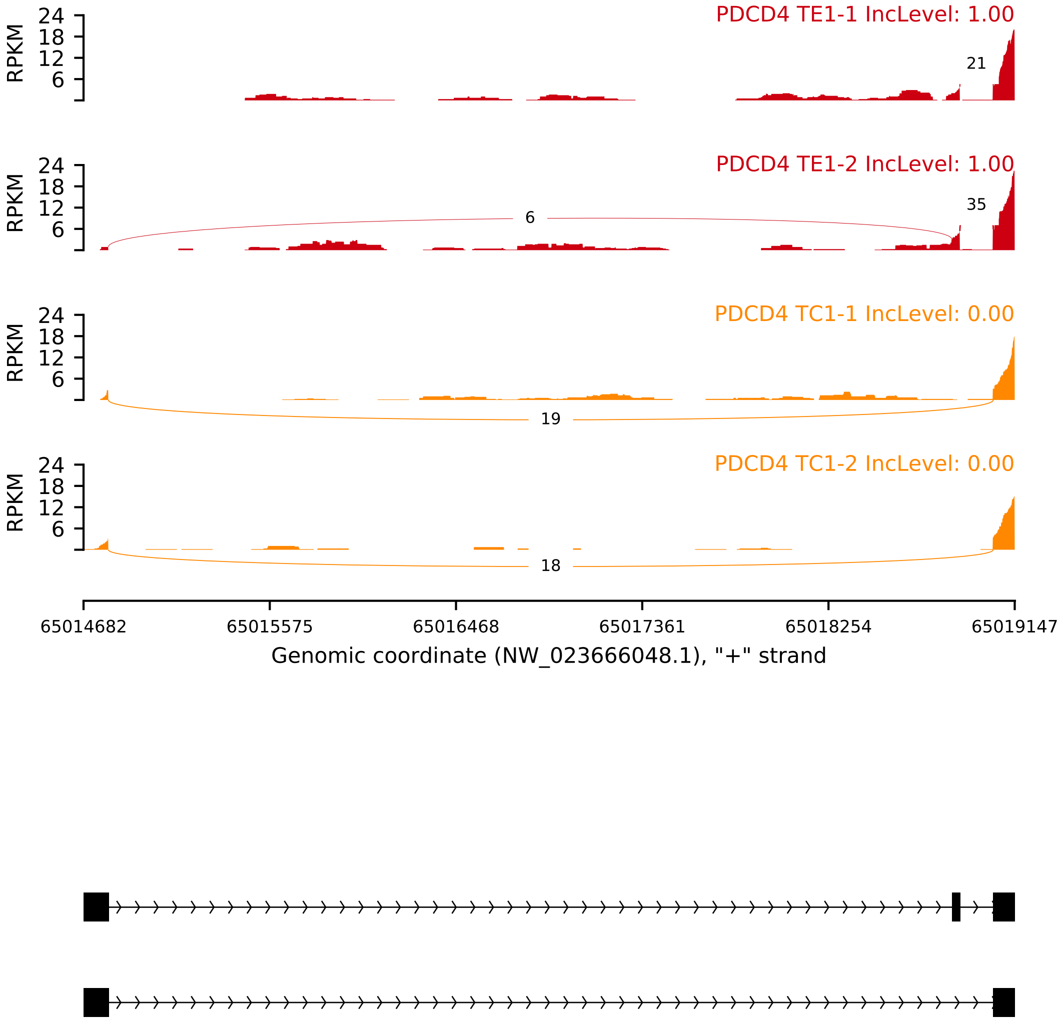

Supplement: Supplementary file 1 — Supplementary Information 1. [file 41598_2022_24687_MOESM1_ESM.zip › raw data/Transcriptomic raw data/7.AS/2.dasplot/TE1vsTC1/SE/4_PDCD4_NW_023666048.1_65014682_65014802_+@NW_023666048.1_65018846_65018885_+@NW_023666048.1_65019044_65019148_+.pdf]
